# Genetic marking and characterization of *Tac2*-expressing neurons in the central and peripheral nervous system

**DOI:** 10.1186/1756-6606-5-3

**Published:** 2012-01-24

**Authors:** Lynn Mar, Fu-Chia Yang, Qiufu Ma

**Affiliations:** 1 Dana-Farber Cancer Institute and Department of Neurobiology, Harvard Medical School, 450 Brookline Ave, Boston, Massachusetts USA 02215

## Abstract

**Background:**

The neurocircuits that process somatic sensory information in the dorsal horn of the spinal cord are still poorly understood, with one reason being the lack of Cre lines for genetically marking or manipulating selective subpopulations of dorsal horn neurons. Here we describe *Tac2-Cre *mice that were generated to express the *Cre *recombinase gene from the *Tac2 *locus. *Tachykinin 2 *(*Tac2*) encodes a neurotransmitter, neurokinin B (NKB).

**Results:**

By crossing *Tac2-Cre *mice with *ROSA26-tdTomato *reporter mice, we directly visualized *Tac2 *lineage neurons in the dorsal root ganglia, the dorsal horn of the spinal cord, and many parts of the brain including the olfactory bulb, cerebral cortex, amygdala, hippocampus, habenula, hypothalamus, and cerebellum. This *Tac2-Cre *allele itself was a null allele for the *Tac2 *gene. Behavioral analyses showed that *Tac2 *homozygous null mice responded normally to a series of algogenic (pain-inducing) and pruritic (itch-inducing) stimuli.

**Conclusions:**

*Tac2-Cre *mice are a useful tool to mark specific subsets of neurons in the sensory ganglia, the dorsal spinal cord, and the brain. These mice can also be used for future genetic manipulations to study the functions of *Tac2*-expressing neurons or the functions of genes expressed in these neurons.

## Background

The spinal cord dorsal horn has long been recognized as the processing center for sensory information including pain, itch, cold, and warmth [1-4]. The dorsal horn is divided into five laminae, which can be identified by lamina-specific innervation by primary sensory fibers and by the expression of molecular markers [3]. For example, neurons in superficial laminae that express the neurokinin 1 receptor (NK1R) and the gastrin-releasing peptide receptor (GRPR) are involved in sensing pain and/or itch [5-7]. Neurons in the inner layer of lamina II that express protein kinase C γ (PKCγ) are the targets of myelinated non-nociceptive afferents [8], and mediate nerve injury-induced mechanical allodynia, a type of pain evoked by innocuous mechanical stimuli [9]. Despite significant progress, the neural circuits that process specific somatic sensory information in the dorsal horn are still not well characterized [3].

Tachykinin peptides are encoded by the *tachykinin 1 *(*Tac1*) and *tachykinin 2 *(*Tac2*) genes and are known to modulate neuronal activity [10]. *Tac1 *encodes a precursor protein that produces two peptides, substance P (SP) and neurokinin A (NKA), whereas *Tac2 *encodes neurokinin B (NKB). Release of SP from sensory neurons in the dorsal root ganglia (DRG) plays a crucial role in modulating pain and itch [11-13]. More specifically, mice lacking *Tac1 *fail to sense moderate to intense thermal and mechanical pain [11]. *Tac2 *expression has also been detected in restricted neuronal populations in the central nervous system [14-16]. In the dorsal spinal cord, for example, *Tac2 *was detected in lamina I and the inner layer of lamina II of both mouse and rat dorsal horns [14-16]. Consistently, it has been suggested that activation of NK3R, the NKB receptor, plays a role in pain modulation [17-19].

The Cre-loxP recombinase system has become a powerful tool to study the morphology and function of specific neuronal populations. An exemplary triumph is the creation of Cre mouse lines that mark distinct subsets of primary sensory neurons, which allowed precise genetic ablation of Cre-expressing sensory neurons, and thus have provided unprecedented insight into the cellular basis of pain and itch [20-23]. The availability of additional precise and neuron-specific Cre lines will allow more such discoveries, especially in light of genome-wide conditional knockout resources [24]. To study the physiological function of *Tac2*, and to characterize *Tac2*-expressing neurons, here we report the generation of *Tac2-Cre *mice in which Cre recombinase is under the control of the *Tac2 *locus, and where *Tac2-Cre *represents a null allele.

## Results

### Generation of *Tac2-Cre *mice

**Figure 1:**
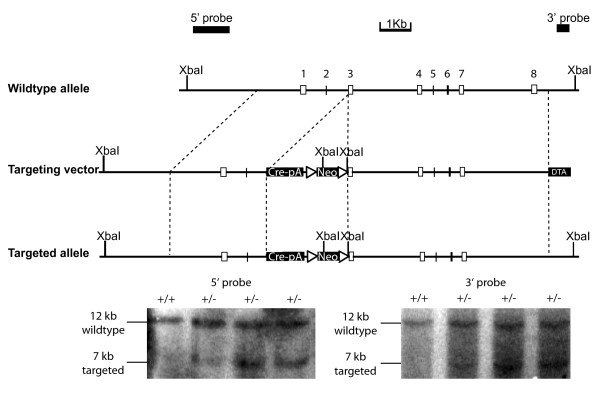
**Generation of *Tac2-Cre *mice**. Top, schematic diagram of the gene targeting strategy used to insert Cre into the wildtype *Tac2 *locus. The *Tac2-Cre *targeting vector included Cre directly downstream of the *Tac2 *ATG codon sequence, an FRT-flanked neomycin positive selection cassette, and a DTA negative selection cassette. XbaI sites and the expected sizes of the resulting DNA fragments are indicated. Below, Southern blot analyses for verification of correct targeting of *Tac2 *locus. Southern blot with XbaI and external probes confirmed correctly targeted homologous recombination events. The 12 kb WT band is seen in all lanes, and two correctly targeted and distinct 7 Kb bands represent the targeted allele at the 5' and 3' homology arms.

The targeting strategy used to generate *Tac2-Cre *mice is illustrated in Figure 1. In the targeting vector, Cre and a neomycin selection cassette were inserted into exon 3, the first coding exon of *Tac2*. The neomycin selection cassette is floxed with *FRT *sites that can be removed later by Flipase-mediated DNA recombination [25]. After electroporation in 129/Sv embryonic stem cells, 192 clones that survived selection with Geneticin were screened via Southern blot analysis, and 9 correctly targeted clones were identified. The digestion of genomic DNA with the restriction enzyme XbaI generated a12Kb fragment for the wildtype allele, and two different 7 Kb fragments for the *Tac2-Cre *mutant allele, depending on whether the 5' or 3' arm external probe was used (Figure 1). Two positive cell lines were used for blastocyst injection and the resulting chimera mice were crossed with C57BL6 wildtype mice. F1 progenies carrying germ line transmission of the *Tac2-Cre *allele were identified, which were then crossed to FLPeR mice to delete the neomycin cassette [25].

### Tac2-Cre expression in the dorsal spinal cord

To analyze the efficiency and fidelity of Cre recombinase activity, *Tac2-Cre *mice were crossed to the Cre-dependent reporter line *ROSA26-loxP-STOP-loxP-tdTomato *[26]. The resulting double heterozygous mice (*Tac2-Cre*; *ROSA26-loxP-STOP-loxP-tdTomato*) will be referred to as *Tac2-Cre*/*Tomato *mice, in which Cre-mediated recombination led to constitutive expression of the red fluorescent protein Tomato. As a result, neurons that expressed *Tac2-Cre*, either transiently or persistently, were permanently marked by Tomato expression.

**Figure 2:**
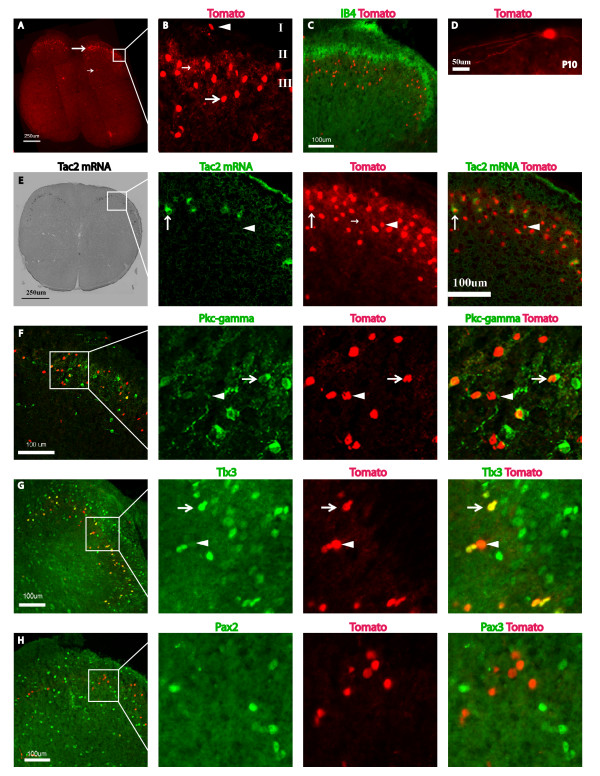
**Characterization Tac2-Cre expressing neurons in the dorsal horn of the spinal cord**. **A: **A transverse section through a P30 spinal cord showing fluorescent Tomato expression enriched in dorsal horn neurons as indicated by the large arrow. Small arrow indicates sporadic Tomato-positive neurons in the deep dorsal horn. **B: **Enlarged inset of the dorsal horn (**A**), showing Tomato-positive neurons in lamina I (arrowhead), as well as the ventral layer of inner lamina II and lamina III (large arrow). Note the local processes in lamina II/III (small arrow). **C: **Double staining of Tomato fluorescence (red) and fluorescently conjugated isolection IB4 (green). Tomato-positive neurons are located ventral to the dorsal layer of inner lamina II marked by IB4-positive sensory terminals. **D: **Transverse section of a P10 spinal cord illustrating a lamina I neuron with long processes. **E: **Co-localization of Tomato and *Tac2 *transcripts. Sections were first photographed for Tomato fluorescence (red), then used to conduct *in situ *hybridization (pseudo-colored green), and the resultant signals were merged. Large arrow indicates a neuron co-expressing Tomato and Tac2 mRNA, while arrowhead indicates one that expressed Tomato but not Tac2 mRNA. Small arrow indicates neuronal processes within inner lamina II and lamina III. **F-H: **Double staining of Tomato (red) and antibodies (green) against PKCγ (**F**), Tlx3 (**G**), and Pax2 (**H**). Arrows mark double-positive neurons and arrowheads mark Tomato-positive neurons that did not co-express PKCγ (**F**) or Tlx3 (**G**).

In the dorsal spinal cord of *Tac2-Cre*/*Tomato *mice, Tomato expression was first detected at postnatal day 4, similar to *in situ *expression analysis ([27], data not shown). By P30, Tomato-positive neurons were observed mainly within the ventral layer of inner lamina II and lamina III (Figure 2A, big arrow; Figure 2B), as they were located ventral to primary sensory terminals labeled by isolectin-B4 (IB4) that innervate the dorsal layer of inner lamina II (Figure 2C) [8]. A few scattered Tomato-expressing cells were also observed in more ventral spinal areas (Figure 2A, small arrow). Furthermore, the processes of these Tomato-positive neurons were short and remained within the narrow laminae (Figure 2B, small arrow; Figure 2E, small arrow), suggesting their role in processing local information. Occasionally, Tomato-expressing neurons were also found in lamina I (Figure 2B, arrowhead), and they exhibited long, transverse processes (Figure 2D). This lamina-specific distribution of *Tac2-Cre*/*Tomato *neurons is consistent with the reported distribution of Tac2 mRNA and NKB peptide [14-16,27].

To further examine efficacy and specificity, we performed double staining of the Tomato protein and Tac2 mRNA, transcribed from the wildtype allele and detected by *in situ *hybridization, on spinal sections of P30 *Tac2-Cre*/*Tomato *heterozygous mice. We found Tomato reporter expression in the majority (169/209, 81%) of neurons with detectable Tac2 transcripts (Figure 2E, arrow), demonstrating that *Tac2-Cre *was able to drive efficient DNA recombination in most *Tac2*-expressing neurons. However, we also found that a significant number of Tomato-expressing dorsal horn neurons failed to show detectable Tac2 mRNA expression (Figure 2E, arrowhead). Notably, the *Tac2*-negative, Tomato-positive neurons intermingled with *Tac2*-positive neurons in the same dorsal horn laminae (Figure 2E). Thus, some dorsal horn neurons expressed *Tac2 *either transiently or at levels below detection using standard *in situ *hybridization techniques, although we cannot rule out the possibility that inserting *Cre *within the *Tac2 *coding sequence led to ectopic expression of *Tac2-Cre*.

Next, we conducted molecular analysis on these Tac2-Cre/Tomato neurons. PKCγ is expressed by neurons within ventral layer of inner lamina II, and 20% (32/160) of Tomato-positive neurons of *Tac2-Cre*/Tomato mice clearly expressed PKCγ (Figure 2F, arrow). The remaining Tomato-expressing neurons were located in areas that exhibited weak PKCγ staining (Figure 2F, arrowhead). This weak PKCγ staining may be indicative of weak PKCγ expressing neurons, or represent axonal or dendritic processes derived from adjacent strong PKCγ-expressing neurons. If the latter is true, this subset of Tomato-positive neurons may not express PKCγ *per se*, but instead connect with PKCγ-expressing neurons.

We reported previously that Tac2 mRNA in the dorsal horn is mainly detected in glutamatergic excitatory neurons marked by the expression of Tlx3, a homeobox protein [27]. Consistent with this, most Tac2-Cre/Tomato-positive neurons (85%, 167/197) continued to express Tlx3 (Figure 2G, arrow) [27,28], and almost none of the Tac2-Cre/Tomato-positive neurons co-expressed *Pax2 *(2%, 2/131), a marker of inhibitory neurons (Figure 2H, green) [28].

### Tac2-Cre/Tomato expression in the brain

**Figure 3:**
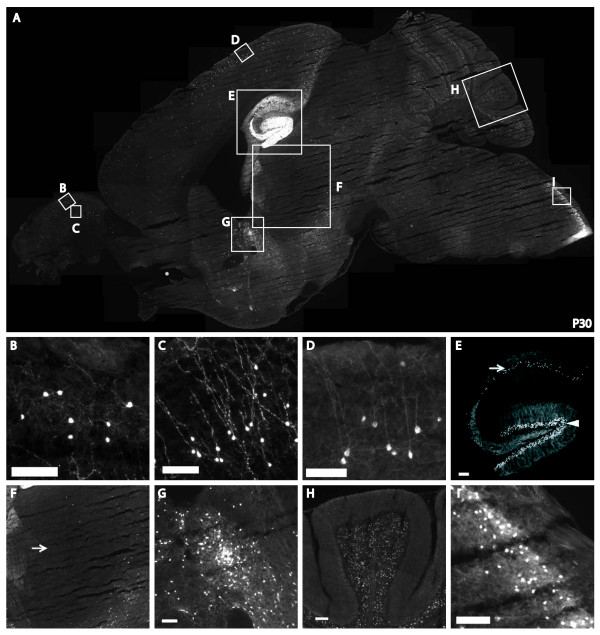
**Tac2-Cre-expressing neurons in multiple brain regions**. A sagittal section of a P30 adult *Tac2-Cre*/*ROSA26-tdTomato *brain (**A**) showing Tomato positive neurons were located within the olfactory bulb (**B**, **C)**, cortex (**D**), hippocampus (**E**), bed nuclei of the stria terminalis (**G**), cerebellum (**H**), medulla (**I**), but not in all the brain regions, such as the thalamus (**F**, arrow). Arrowhead and arrow in **E **indicate the dentate gyrus and the CA1-CA3 regions of the hippocampus, respectively. Scale bar, 50 um.

**Figure 4:**
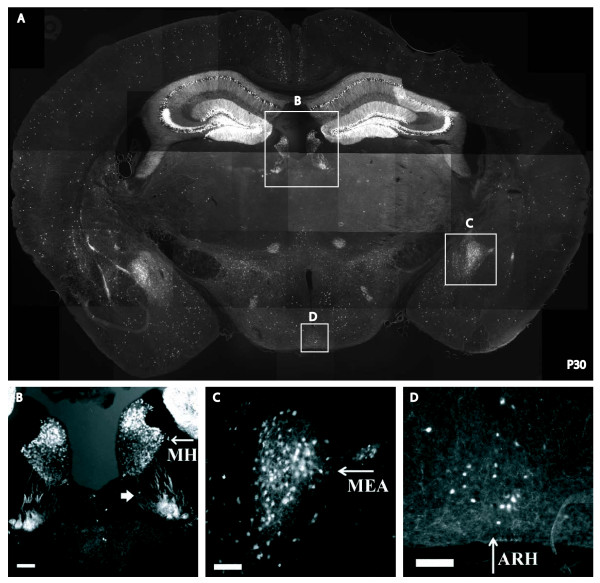
**Tac2-Cre neurons located in additional brain regions**. A transverse section of a P30 adult *Tac2-Cre*/*ROSA26-tdTomato *brain showing Tomato-positive neurons (**A**). Note Tomato expression within the medial habenula (**B**, "MH"), with processes projecting ventral laterally (**B**, arrowhead). Tomato expression was also detected in the medial amygdalar nucleus (**C**, "MEA"), and the arcuate hypothalamic nucleus (**D**, "ARH"). Scale bar, 50 um.

Tomato-positive neurons were detected in multiple brain regions as shown in a sagittal brain section of a P30 *Tac2-Cre*/*Tomato *mouse (Figure 3), and this result is largely consistent with previous expression analyses [14,15]. Within the main olfactory bulb, Tomato labeled neurons were located within the periglomerular layer (Figure 3A, B) and the granule layer (Figure 3A, C). Notably, labeled neurons in the granule layer sent long apical dendrites (Figure 3C), a morphology typical of inhibitory granule cells as reviewed by Shepherd *et al*. [29]. In the cerebral cortex, Tomato fluorescence was enriched in layers II and III, but was also found scattered through other cortical layers (Figure 3A, D). These neurons showed pyramidal shaped soma with long dendrites toward the apical surface (Figure 3D), a morphology typical of glutamatergic projection neurons [30]. In the hippocampus, Tomato was expressed in the granule cell layer of the dentate gyrus and the pyramidal cell layer of the CA1-CA3 regions (Figure 3A, E, Figure 4A). Other Tomato-expressing neurons were observed in the bed nuclei of the stria terminalis (Figure 3A, G), the granule cells in the cerebellum (Figure 3A, H), and the superficial laminae of the spinal trigeminal nuclei (Figure 3A, I). Furthermore, a coronal section demonstrated Tomato expression in the medial habenula (Figure 4A, B), amygdala nuclei (Figure 4A, C), and scattered neurons in hypothalamic nuclei including the arcuate nucleus (Figure 4A, D). In contrast, Tomato expression was rarely detected in other brain regions such as the thalamus (Figure 3A, F).

### Tac2-Cre/Tomato expression in the peripheral nervous system

**Figure 5:**
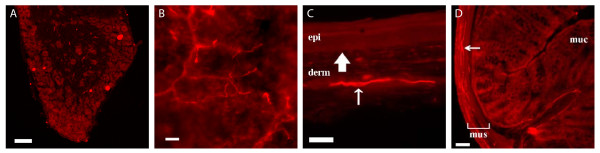
**Tac2-Cre-expressing neurons in the DRG and their innervation of peripheral targets**. Adult *Tac2-Cre*/*ROSA26-tdTomato *mice showed fluorescence labeling in a small subset of lumbar DRG neurons (**A**). Their primary afferents innervated the glabrous skin in a wholemount preparation (**B**), and a transverse section showed selective innervation within the dermal layer (**C**, arrow). "derm": dermis and "epi": epidermis, and the solid arrow indicates boundary between dermis and epidermis. In a transverse section of the colon, Tomato-positive fibers were detected in the muscle layer (**D**, "mus"), by not in the mucousal layer (**D**, "muc").

Within the peripheral nervous system, Tac2-Cre/Tomato was detected in a small subset of neurons in the DRG (Figure 5A) and in the sympathetic ganglia (data not shown). This is surprising because detection of *Tac2 *mRNA in adult DRG has not yet been reported ([10,14], data not shown). Given Tomato reporter expression was present at P0 (data not shown), these neurons might express *Tac2 *transiently during embryonic development. Interestingly, these Tomato-positive neurons innervated selective peripheral targets (Figure 5B-D). Wholemount preparations revealed Tomato-positive fibers in the glabrous skin (Figure 5B), and as shown in a transverse section, these fibers were located within the dermal layer (Figure 5C). In the colon, a visceral organ, thin circumferential Tomato-positive fibers were detected in the muscle layer (Figure 5D), and *Tac2-Cre *expression was also detected in the bladder (data not shown).

### *Tac2 *null mice responded normally to pain-related or itch-related stimuli

**Figure 6:**
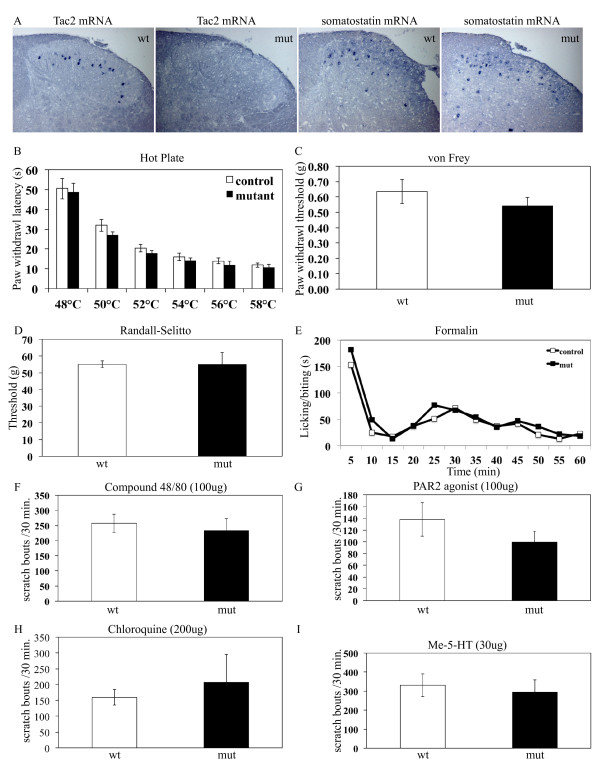
***Tac2 *Mutant mice did not exhibit defects in pain and itch sensation**. **A: ***In situ *hybridization analyses of wildtype and mutant spinal cords using *Tac2 *and *somatostatin *antisense probes demonstrated the loss of *Tac2 *expression in mutant mice, while the expression of the somatostatin neuropeptide gene was unaffected. **B-I: **Responses to nociceptive and pruritic stimuli were normal in homozygous *Tac2-Cre *mice. Mutant (mut) *Tac2-Cre *mice were indistinguishable from wildtype (wt) littermates in terms of latency of hindpaw withdrawal upon application of various noxious temperatures (**B**, *p *= 0.19, 0.33, 0.09, 0.71, 0.39, 0.40; 3 female controls, 3 male controls, 4 female mutants, 3 male mutants); in mechanical threshold for eliciting hindpaw withdrawal following application of graded pressure by von Frey fibers (**C**, *p *= 0.48; 12 female controls, 7 male controls, 9 female mutants, 12 male mutants), and to the tail by the Randall-Selitto apparatus (**D**, *p *= 1.0; 3 female controls, 2 female mutants, 2 male mutants). Licking and biting responses to hindpaw formalin injections were unaffected in the first phase, the first 10 minutes post-injection, and the second phase, 10-60 minutes post-injection (**E**, *p *= 0.32; 4 female controls, 3 male controls, 5 female mutants, 2 male mutants). Furthermore, there was no significant difference in itch-related scratching responses following administration at the indicated dosages of pruritic agents including Compound 48/80 (**F**, *p *= 0.65; 4 female controls, 1 male control, 4 female mutants, 4 male mutants); PAR2 agonist (**G ***n *= 8; *p *= 0.29; 8 female controls, 8 male controls, 8 female mutants, 8 male mutants); Chloroquine (**H**, *p *= 0.64; 4 female controls, 4 female mutants, 3 male mutants); and Me-5-HT (**I**, *p *= 0.33; 8 female controls, 8 male controls, 8 female mutants, 8 male mutants).

In *Tac2-Cre *mice, the Cre expression cassette, which contained the HSV thymidine kinase polyA signal, was inserted directly after the first ATG codon of the *Tac2 *gene, thereby creating a null allele. Consistent with this, we demonstrated a loss of *Tac2 *expression in the dorsal spinal cord of mutant mice (Figure 6A). In contrast, a control *in situ *hybridization using the somatostatin probe showed comparable signals in mutant versus control littermates (Figure 6A). Tachykinin peptides, such as SP and NKA, have been implicated in modulating pain and itch [11-13,31]. Therefore we sought to determine if *Tac2 *also played a role in modulating pain and/or itch sensation. *Tac2*^Cre/Cre ^homozygous null mice survived at the expected Mendelian ratio. We performed a series of pain and itch behavior assays on these mutant mice, using wildtype or *Tac2*^Cre/+ ^heterozygous littermates as controls. In comparison to control mice, *Tac2*^Cre/Cre ^homozygous null mice responded normally to painful stimuli, including noxious heat (48°C, 50°C, 52°C, 54°C, 56°C, 58°C, Figure 6B), light mechanical stimuli (von Frey assay, Figure 6C), intense mechanical stimuli (Randall-Selitto; Figure 6D), and chemical stimuli (formalin, Figure 6E). Moreover, like control littermates, *Tac2*^Cre/Cre ^homozygous null mice showed similar scratching responses following nape injection of itching compounds [23,32,33], including Compound 48/80 (Figure 6F), PAR2 agonist (Figure 6G), chloroquine (Figure 6H), and serotonin (Figure 6I).

## Discussion

We have generated a *Tac2-Cre *knock-in mouse line that drives Cre recombinase expression in various parts of the nervous system, including sensory neurons innervating skin dermis and visceral organs, a subset of excitatory neurons in the dorsal spinal cord, interneurons in the olfactory bulb, projection neurons in the cerebral cortex, neurons in specific amygdala nuclei, granule cells in the dentate gyrus, pyramidal neurons in the hippocampal CA1-CA3 regions, granule cells in the cerebellum, and others. Thus, this Cre line will be of broad utility to the neuroscience community.

In the dorsal spinal cord, Tac2-Cre is expressed in the ventral layer of inner lamina II and lamina III, and occasionally in lamina I and in more ventral spinal areas (Figure 2A, B). *Tac2-Cre *neurons in the dorsal horn are excitatory in nature and are associated with strong or weak PKCγ expression. Overall, Cre expression in *Tac2-Cre *mice is precise and efficient in those neurons with persistent *Tac2 *expression, as demonstrated by double staining results (Figure 2B). However, the lineage tracing experiments also revealed a set of spinal neurons and peripheral sensory neurons that might express *Tac2 *transiently.

The expression pattern of Tomato in the dorsal horn of *Tac2-Cre*/*Tomato *mice is largely consistent with existing literature, but also differed in a few of respects from the NKB immunostaining pattern observed in the rat dorsal horn [16]. For example, whereas many NKB-immunostained neurons were observed in dorsal horn lamina I in rat [16], Tomato-positive neurons were only occasionally seen in lamina I of *Tac2-Cre*/*Tomato *mice (Figure 2B), consistent with *in situ *expression analyses [14,27]. This discrepancy could be due to species difference and/or the binding of the NKB antibody to certain NKB-negative neurons.

One notable feature of this *Tac2-Cre *mouse line, at least in the dorsal spinal cord, is the post-natal onset of Tac2-Cre expression (from P4-P10). Consequently, conditional knockouts in combination with this Cre line will be useful for studying the roles of interested genes in controlling postnatal development of neural circuits or controlling physiological functions of dorsal horn neurons.

Several recent genetic marking studies have led to identification of somatic sensory neurons that innervate specific peripheral targets. For example, *MrgprD*-expressing polymodal nociceptors, representing 30% of DRG neurons, innervate exclusively skin epidermis [34,35]. Similarly, MrgprB4-expressing neurons innervate skin epidermis around the hairs [36]. To our knowledge, *Tac2-Cre*/*Tomato *may represent the first molecular marker of a small subset of DRG neurons that innervate selective deep tissues, such as the dermis and visceral organs.

Behavioral studies suggested that the loss of *Tac2 *did not result in a detectable effect on pain or itch sensation, at least based on the assays used in this study. This finding is surprising considering that intrathecal injection of NKB or NK3R agonist has been reported to cause hyperalgesia [18,19] or hypoalgesia [17]. However, it is consistent with observations that no obvious pain deficits had been reported in NK3 knockout mice [37-39]. Perhaps the lack of behavioral deficit in *Tac2 *null mice is the result of redundant function with SP and NKA. Future studies on mice that lack both *Tac1 *and *Tac2 *could be informative in this regard.

## Conclusions

The *Tac2-Cre *knock-in mouse line is able to mark Tac2 lineage neurons in the dorsal root ganglia, the dorsal spinal cord and various parts of the brain. Thus, this Cre line will be a useful genetic tool for conditionally manipulating genes in selective populations of neurons.

## Methods

### Generation of *Tac2-Cre *targeting Construct

A 10 Kb EagI/XbaI fragment, containing all 8 exons of the *Tac2 *gene, was subcloned from the BAC RP23-270B22 (UCSC *Mus musculus *Genome Brower). Next, the *Cre *coding sequence with a polyA signal was inserted in-frame with the first ATG of the *Tac2 *coding sequence. Finally, an *FRT *flanked neomycin cassette was inserted downstream of the *Cre *cassette. The orientation and sequence of the targeting construct was confirmed by sequencing.

### Generation of *Tac2-Cre *mice

The targeting construct was linearized using NotI and electroporated into 129/Sv embryonic stem (ES) cells. ES cells were drug selected using geneticin and correctly targeted clones were identified using Southern blot analysis. Genomic DNA was extracted from 96 well colonies, digested with XbaI, Southern blotted, and hybridized using both 5' and 3' arm external probes. Correctly targeted *Tac2 *alleles exhibited two distinct 7 kb targeted bands, in contrast to the 12 Kb wildtype band. Correctly targeted clones were used by the Dana-Farber ES cell core facility to generate transgenic mice via blastocyst injection. After establishing germ-line transmission, the neomycin cassette was excised by crossing to an *FLP *deleter strain [25] to generate the *Tac2-Cre *mouse line. Genotyping for the *Tac2-Cre *wildtype and mutant alleles was performed using PCR with *Tac2 *sense, *Tac2 *antisense, and Cre primers. Primer sequences were as follows: *Tac2 *sense primer (5' - atccaacgctcttgagatcagggctcagat - 3'), *Tac2 *antisense primer (5'-ggacatcttaccttactgag-3'), Cre primer (5'-tcgaccggtaatgcaggcaa-3'). The product sizes were as follows: wildtype allele, *Tac2 *sense and antisense: 256 bp; mutant allele, *Tac2 *sense and Cre: 216 bp. Products were electrophoresed on 1.5% agarose gels, stained with ethidium bromide and photographed.

### Animals

Heterozygous or homozygous *Tac2-Cre *mice were crossed to heterozygous or homozygous *ROSA26-tdTomato *mice (Madisen et al., 2010). Offspring were genotyped by PCR for *Tac2-Cre *and *ROSA26-tdTomato *alleles. Double heterozygous *Tac2-Cre *and *ROSA26-tdTomato *mice at P30 were used in immunohistochemical studies. Eight-week-old homozygous *Tac2-Cre *and control littermate mice were used in behavior analyses. The Institutional Animal Care and Use Committee at Dana-Farber Cancer Institute approved all experimental and behavioral test procedures.

### *In situ *hybridization (ISH) and Immunostaining

ISH procedure of the *Tac2 *probe was previously described [27,40]. Briefly, mice were deeply anesthetized and perfused through the left cardiac ventricle with a fixative containing 4% freshly depolymerized paraformaldehyde (4% PFA). Spinal cords were dissected and fixed overnight in 4% PFA, equilibrated overnight with 20% sucrose, and frozen in OCT (O.C.T. Compounds, BDH) on dry ice. 12 uM sections were prepared and gently permeated by Proteinase K, treated with triethanolamine, and hybridized with 1-2 μg/mL of DIG labeled antisense probe for over 12 hours. Non-specific binding was washed away by 0.2xSSC stringency at 60°C, blocked by sheep serum and incubated with Alkaline phosphatase conjugated anti-DIG antibody. Alkaline phosphate reaction was catalyzed by NBT and BCIP (Roche). For tdTomato/ISH double staining, the Tomato fluorescent signal was directly photographed followed by performing ISH. The ISH signals were pseudocolored and then overlayed with the Tomato signal.

### Immunohistochemical Analyses

Tissues were collected, fixed, and frozen as for ISH. 12 μm cryosections were dried at room temperature for 30 min and then fixed with 4% PBS-buffered paraformaldehyde solution for 10 min on ice. After three washes in PBS, sections were incubated for 30 min in 10% goat serum diluted in PBS containing 0.3% Triton X-100 (PBST), and for 1 h at room temperature in 1:200 biotin conjugated IB4 (10 mg/ml, Sigma, USA) or 1:1000 dilution of a rabbit αPKCγ antibody (SC-211, Santa Cruz), a 1:1000 dilution of a guinea pig αTlx3 antibody (a gift from C. Birchmeier, Germany), or a 1:1000 dilution of a rabbit α-Pax2 antibody (SC-211, Santa Cruz). Following three washes in PBST, the sections were incubated for 1 h in a 1:200 dilution of Alexa Fluor 488-Streptavidin (Invitrogen), Alexa Fluor 488-Goat-α-Guinea Pig (Invitrogen), or Alexa Fluor 488-Goat α-Rabbit (A11034, Invitrogen). After three washes in PBST, the sections were mounted in 90% glycerol, 0.5% N-propyl gallate (pH 8.0) and analyzed using a fluorescent microscope.

### Behavioral measurements

The experimenter was blind to which group an animal belonged.

Hot Plate test: naïve, awake, and unrestrained mice were individually removed from their home cage and placed on a metal surface pre-heated and maintained at the indicated temperatures (IITC Model PE34M-HC). The mice were enclosed within transparent 20 cm high walls, in a 14 × 14 cm area Plexiglas chamber. The latency to performing a hindpaw flutter/shake, lick, or jump (whichever occurred first) was measured to the nearest 0.1 s. The cut-off time for 48-52°C was 60 seconds, while for 54-58°C it was 30 seconds.

von Frey threshold: awake, unrestrained, and acclimatized mice were individually removed from their home cage and habituated for 30 min in a metal mesh enclosure for 2 days. Mechanical sensitivity threshold of the stimulated hindpaw was measured for the next 2 consecutive days. Mechanical sensitivity was measured by determining the median 50% foot withdrawal threshold (measured in mN of discrete bending force), when a single prick to the hindfoot was applied steadily (>1 s duration) with a von Frey monofilament via the up-down method.

Randall-Selitto: A Randall-Selitto apparatus (Ugo Basile, Italy) was used to measure the withdrawal threshold. The animals were restrained in plastic enclosures and treated gently during the experiments. A cone-shaped pusher with a rounded tip (diameter of the base: 9 mm) was applied to the tail. The intensity of pressure causing an escape reaction was defined as the withdrawal threshold. Measurements were performed 3 times at intervals of several minutes, and the mean value was taken as the threshold.

Formalin test: awake, unrestrained, and acclimatized mice were individually removed from their home cage and habituated for 30 min in mesh enclosures. Then, 25 μL of 5% formalin was injected subcutaneously into the plantar surface of the right hindpaw using a 0.5 mL insulin syringe with a 28G1/2 needle. Mice were then returned to the mesh enclosure, and behavioral observations were video taped. Video recording was subsequently played back, and the total time spent licking and/or biting the right hindpaw over the next 60 minutes was recorded to the nearest second in 5-min blocks. The early/acute phase was defined as 0-10 min post-injection, and the late/tonic phase as 10-60 min post-injection.

Itch assay: awake and unrestrained mice were individually removed from their home cage and acclimatized for 1 hour daily, for 5 continuous days, in Plexiglas containers (10 × 10 × 12 cm) atop a glass floor. At least two days before the injections, the nape of the neck (approximately 15 × 10 mm area) was shaved after brief anesthesia with isoflurane (2% in 100% oxygen). During the experiment, a camcorder (Sony Model DCR-SR220) was positioned to video-record the scratching behavior of mice. 100 μg of Compound 48/80 (Sigma-Aldrich), 100 μg of PAR2 agonist SLIGRL-NH_2 _(Bachem), or 30 μg of serotonin agonist α-Me-5-HT (Tocris) in 50 μl of sterile saline was injected intradermally into the nape using a 0.5 ml insulin syringe with a 28G1/2 needle (Becton-Dickinson). On the day of the experiment, mice were placed in containers and habituated for 15 min. Then, the behavior of the mice was video recorded 30 min before (baseline behavior), and 30 min after, the chemical was injected. The experimenter was present briefly - once to start the video recording, and once later to administer the chemical. The video recording was subsequently played back and scratching bouts or wipes were counted. A scratching bout was initiated by lifting of the hind paw to the region to be scratched, and ended when the hind paw was returned to the floor or to the mouth for licking. Only the bouts or wipes to the shaved region were counted.

### Statistical Analyses of Pain and Itch Behaviors

All data are presented as mean +/- SEM. Hot plate, von Frey, and Randall-Selitto data was calculated as the average of two independent tests performed on two consecutive days. For formalin and itching behaviors, the mean numbers of licking/biting and scratching bouts, respectively, and SEM during the period were calculated for each group. The difference between the mutant and control group was subjected to a Student's t test (two-sample assuming unequal variance), with p < 0.05 considered statistically significant.

## Competing interests

The authors declare that they have no competing interests.

## Authors' contributions

QM conceived and supervised the conduct of the study. LM generated the *Tac2-Cre *mice and carried out all the molecular and behavioral analyses. LM and FCY carried out peripheral innervation analyses. LM and QM analyzed the molecular and behavioral data. LM and QM drafted the article, with help in editing from FCY. All authors read and approved the final manuscript.
